# FoxM1 drives ADAM17/EGFR activation loop to promote mesenchymal transition in glioblastoma

**DOI:** 10.1038/s41419-018-0482-4

**Published:** 2018-04-27

**Authors:** Chunli Zhang, Xiu Han, Xiao Xu, Zhengrong Zhou, Xi Chen, Yu Tang, Jie Cheng, Nida Fatima Moazzam, Fei Liu, Jing Xu, Wanxin Peng, Fengyi Du, Bin Zhang, Zhiwen Song, Jian Zeng, Aihua Gong

**Affiliations:** 1 Department of Cell Biology, School of Medicine, Jiangsu University, Zhenjiang, 212013 Jiangsu China; 2 Department of Clinical Laboratory, Maternal and Child Health Hospital of Jiading District, Shanghai, 201821 China; 3 Department of Laboratory Medicine, Affiliated Hospital of Jining Medical University, Jining, 272000 Shandong P. R. China; 4 Department of Orthopedics, The Third Affiliated Hospital, Soochow University, Changzhou, 213003 Jiangsu China

## Abstract

Mesenchymal transition (MES transition) is a hallmark of glioblastoma multiforme (GBM), however, the mechanism regulating the process remains to be elucidated. Here we report that FoxM1 drives ADAM17/EGFR activation loop to promote MES transition in GBM. Firstly, FoxM1 expression was positively associated with ADAM17 expression, and their expression was correlated with the mesenchymal features and overall patient survival of GBM. Overexpressing FoxM1 or ADAM17 increased the mesenchymal phenotype of glioma cells, which could be reversed by silencing FoxM1 or ADAM17. Importantly, FoxM1 bound to the ADAM17 promoter to transcriptionally upregulate its expression. Using gain- and loss-of-function studies, we showed that FoxM1/ADAM17 axis promoted the MES transition in glioma cells. Moreover, tissue microarray analysis and orthotopic xenograft model further confirmed that FoxM1/ADAM17 axis played key roles in malignancy of GBM. Mechanistically, FoxM1/ADAM17 axis activated the EGFR/AKT/GSK3β signaling pathway and ADAM17/EGFR/GSK3β axis could maintain FoxM1 stability in glioma cells. Taken together, our study demonstrated that FoxM1/ADAM17 feedback loop controlled the MES transition and regulated the progression of GBM, raising the possibility that deregulation of this loop might improve the durability of therapies in GBM.

## Introduction

GBM is the most common malignant primary brain tumor in adults^1,2^. Integrated genomic analyses enable the molecular classification of GBM into neural, proneural, classical and mesenchymal subtypes^3,4^. GBM patients in the mesenchymal subtype exhibit radio- and chemo-resistant signature, increased invasiveness, and relatively worse prognosis than proneural subtype^4–6^. Moreover, recurrences and radio-resistance are associated with the mesenchymal shift in GBM^2^. It is established that collaboration among transcription factors^6–10^, multiple signaling pathways^11–14^ and other molecules^10,15^ are involved in mesenchymal shift in GBM. All these suggest that MES transition might be of great relevance of GBM progression. Therefore, it is critical to elucidate the molecular mechanisms underlying the MES transition in GBM.

FoxM1 is a proliferation-specific transcriptional factor and is of great importance in regulating G1–S and G2–M phase of the cell cycle and mitotic spindle integrity^16–18^. In a wide range of cancers, including GBM, elevated expression of FoxM1 is well recognized and it is strongly linked to tumor malignancy, angiogenesis, and invasiveness^19–24^. Of note, Zhang et al. demonstrated that direct FoxM1–β-catenin interaction enhanced β-catenin expression and Wnt signaling, which aids in developing tumorigenesis in glioma^25^. Additionally, our previous studies showed that FoxM1 upregulated expression of PDGF-A and STAT3 to maintain the self-renewal and tumorigenicitiy of glioma stem-like cells^26^. Ours and others studies have provided compelling evidence that FoxM1 played critical roles in glioma progression. However, the role of FoxM1 in regulating the MES transition in glioma is still unclear.

A disintegrin and metalloproteinase 17 (ADAM17), also known as tumor necrosis factor-alpha converting enzyme (TACE), is a membrane-bound enzyme that cleaves cell surface proteins, such as cytokines (e.g. TNF-α), cytokinereceptors (e.g. IL-6R and TNF-R), ligands of ErbB (e.g. TGF-α and amphiregulin) and adhesion proteins (e.g. Lselectin and ICAM-1)^27–29^. ADAM17 plays an important role in inflammation and cancer progression. Some recent studies have shown that ADAM17 overexpression was correlated with high tumor grade and poor prognosis in glioma patients^30–33^. However, it remains to be identified whether ADAM17 comes into play in the MES transition in GBM. Additionally, Affymetrix analysis and RT-PCR demonstrated that the FoxM1 –/– lungs displayed a 90% reduction in the expression level of ADAM17^17^, suggesting that FoxM1 might regulate ADAM17 expression, however, the underling mechanism remained to be elucidated.

In this study, we established the direct link between FoxM1 and ADAM17, and verified their roles in MES transition in GBM. Mechanistically, we confirmed that FoxM1/ADAM17 axis activated EGFR/AKT/GSK3β pathway, and then stabilized the FoxM1 protein expression. Furthermore, FoxM1/ADAM17 axis promoted the tumorigenicity of glioma cells and the progression of GBM. Collectively, it is the first time to report that the FoxM1/ADAM17 feedback loop promotes the MES transition in GBM.

## Results

### The expression profiles of FoxM1 and ADAM17 are positively correlated with mesenchymal features in GBM

**Fig. 1:**
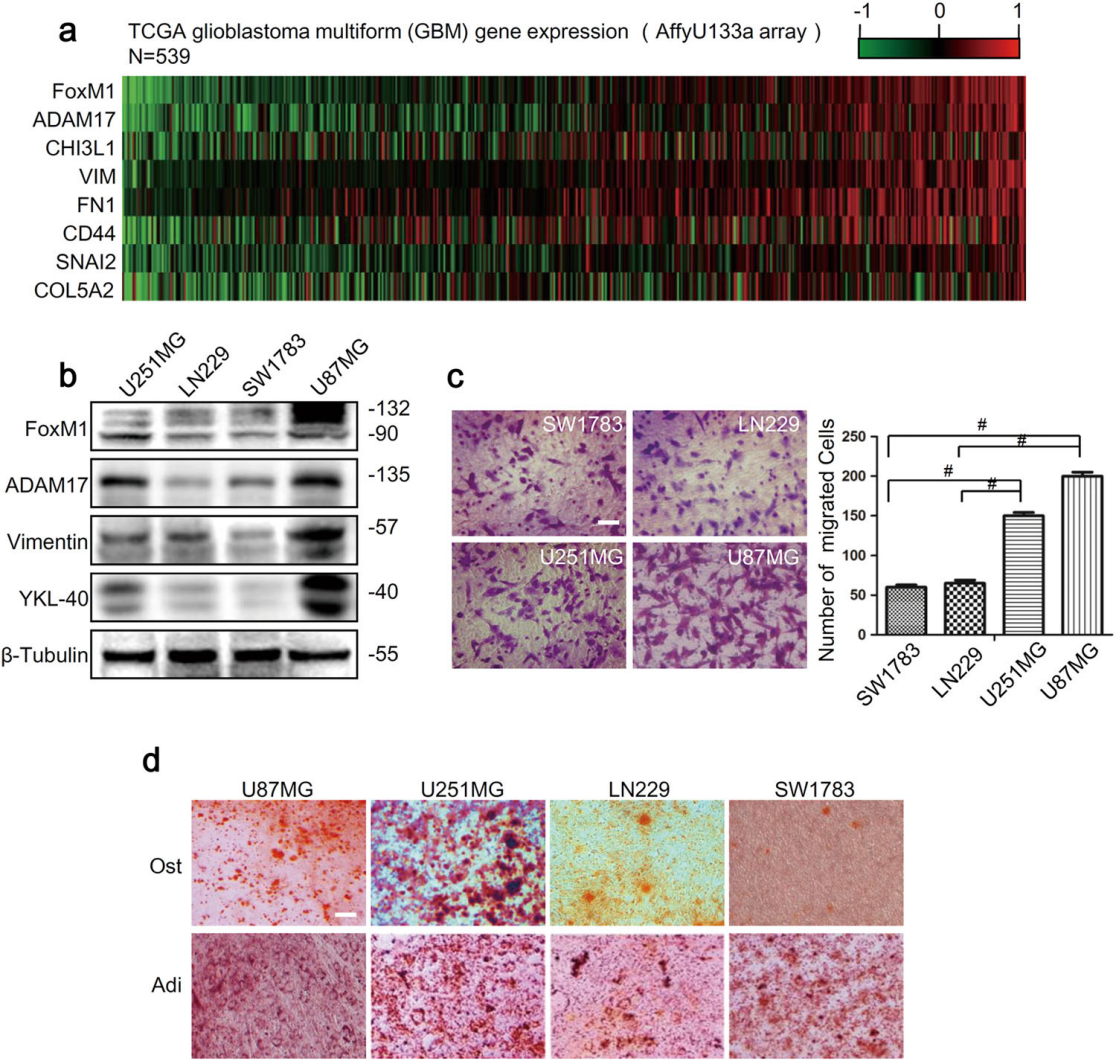
The expression profiles of FoxM1 and ADAM17 are positively correlated with mesenchymal features in GBM. **a** Heatmap of the gene expression profile revealed that FoxM1 and ADAM17 expression profiles were associated with mesenchymal markers expression in glioma specimens from TCGA database. **b** FoxM1, ADAM17 and mesenchymal markers expression in glioma cells were detected by western blot. β-Tubulin was used as a loading control. **c** The transwell migration assay was conducted to count the migrated cells of four glioma cell lines. Scale bar = 100 μm. The data are shown as the mean ± s.d. of three independent experiments. Student’s *t* test was used to determine the significance of the differences between the groups (^#^*P* < 0.0001, Student’s *t* test)**. d** The conditional medium was used to investigate the osteogenesis and adipogenesis potential of four glioma cell lines. Scale bar = 100 μm. Ost osteogenesis, Adi adipogenesis

To investigate the association of FoxM1 and ADAM17 with the MES phenotype, we first analyzed the expression levels of FoxM1, ADAM17 and mesenchymal markers in glioma specimens from The Cancer Genome Atlas (TCGA). Gene expression heat maps and correlation analysis revealed that the expression of ADAM17 was highly associated with that of FoxM1, meanwhile both of them were correlated with the expression of mesenchymal markers in GBM (Fig.1a, S1a). Moreover, Patients with high expression of FoxM1 had a median survival of 289.5 days as compared with 407 days for the patients with low expression of FoxM1(*P* = 0.0232), and patients with high expression of ADAM17 had a median survival of 199.5 days as compared with 404 days for the patients with low expression of ADAM17(*P* = 0.009). The corresponding Kaplan–Meier survival curves showed poor survival rate in group with high FoxM1 or ADAM17 expression (Supplementary Fig. S1b). Subsequently, we analyzed the expression levels of FoxM1, ADAM17 and mesenchymal markers (vimentin, YKL-40) in four glioma cell lines. We noticed that the expression of FoxM1, ADAM17 and mesenchymal markers were relatively higher in U251MG and U87MG cells compared with those in SW1783 and LN229 cells (Fig.1b, Supplementary Fig S1c). Moreover, the immunofluorescence analyses showed that the expression of FoxM1, ADAM17 and mesenchymal markers were higher in U87MG cells compared to those in SW1783 cells (Supplementary Fig. S1d). The findings suggested that FoxM1 and ADAM17 expression were correlated with each other and both of them were associated with mesenchymal states in GBM.

To further confirm the biology behavior of glioma cells, we conducted transwell migration assay and adipogenic, osteogenic differentiation assays. The results showed that the numbers of migrated cells in U251MG and U87MG cells were more than those in SW1783 and LN229 cells (Fig.1c). Meanwhile, under the adipogenic treatment, U251MG and U87MG cells formed oil red-O-positive cells with cytoplasmic accumulation of lipid vacuoles and eccentric deviation of nuclease within cells. In contrast, SW1783 and LN229 cells only showed weak staining (Fig.1d). In osteogenic differentiation assay, the alkaline phosphatase–positive dots were observed more in U251MG and U87MG cells than those in SW1783 and LN229 cells (Fig. 1d). These suggested that U251MG and U87MG cells had much stronger abilities of migration, adipogenesis and osteogenesis than in SW1783 and LN229 cells. Taken together, it was confirmed that the expression profiles of FoxM1 and ADAM17 were positive correlated with mesenchymal phenotype in GBM (Tables 1 and 2).

**Table 1:** The primers for PCR

Name	Sequence (5′ to 3′)
FoxM1 RT-Forward	TGGACCAGGTGTTTAAGCAGC
FoxM1 RT-Reverse	GGGAGTTCGGTTTTGATGGTC
ADAM17 RT-Forward	TTATTGGTGGTAGCAGAT
ADAM17 RT-Reverse	AAGTGTTCCGATAGATGT
vimentin RT-Forward	CGGGAGAAATTGCAGGAGGA
vimentin RT-Reverse	AAGGTCAAGACGTGCCAGAG
YKL-40 RT-Forward	TCCAGTGCTGCTCTGCATAC
YKL-40 RT-Reverse	CCAGGTGTCGATGTGATCGT
GAPDH RT-Forward	GCTCAGAACACCTATGGGGA
GAPDH RT-Reverse	AAGTGTTCCGATAGATGT
ADAM17 promoter CHIP-Forward	AATGGACCAAGTATAGGATT
ADAM17 promoter CHIP- Reverse	GCAGACACTTCAACAAAT

**Table 2:** shRNA target sequences and mutant primer sequences

Name	Sequence(5’ to 3’)
target sequences of sh-EGFP	TACAACAGCCACAA CGTCTAT
target sequences of sh-FoxM1	GCCAATCGTTCTCTGACAGAA
target sequences of sh-ADAM17	CCTATGTCGATGCTGAACAAA
ADAM17 promoter Forward	AATGGACCAAGTATAGGATT
ADAM17 promoter Reverse	GCAGACACTTCAACAAAT
ADAM17 m1 Forward	GCATAAAACAGCAACATGTACCCAACGT
ADAM17 m1 Reverse	AGTTCAACCAAAGTTTATAAAAGTAGGACATAAT
ADAM17 m2 Forward	GCCATTCACTAAACTATACAGAGCT
ADAM17 m2 Reverse	TATGGTACTGAATAAAACAGTCTAAAAAC
ADAM17 m3 Forward	TATGGTACTGAATAAAACAGTCTAAAAAC
ADAM17 m3 Reverse	TATGGTACTGAATAAAACAGTCTAAAAAC
ADAM17 m4 Forward	GGGGCGTGGAGCAAATGTGCATT
ADAM17 m4 Reverse	TGCTGTAGGGAGAGGGTCTGCAGACACT

### FoxM1 promotes MES transition in glioma cells

**Fig. 2:**
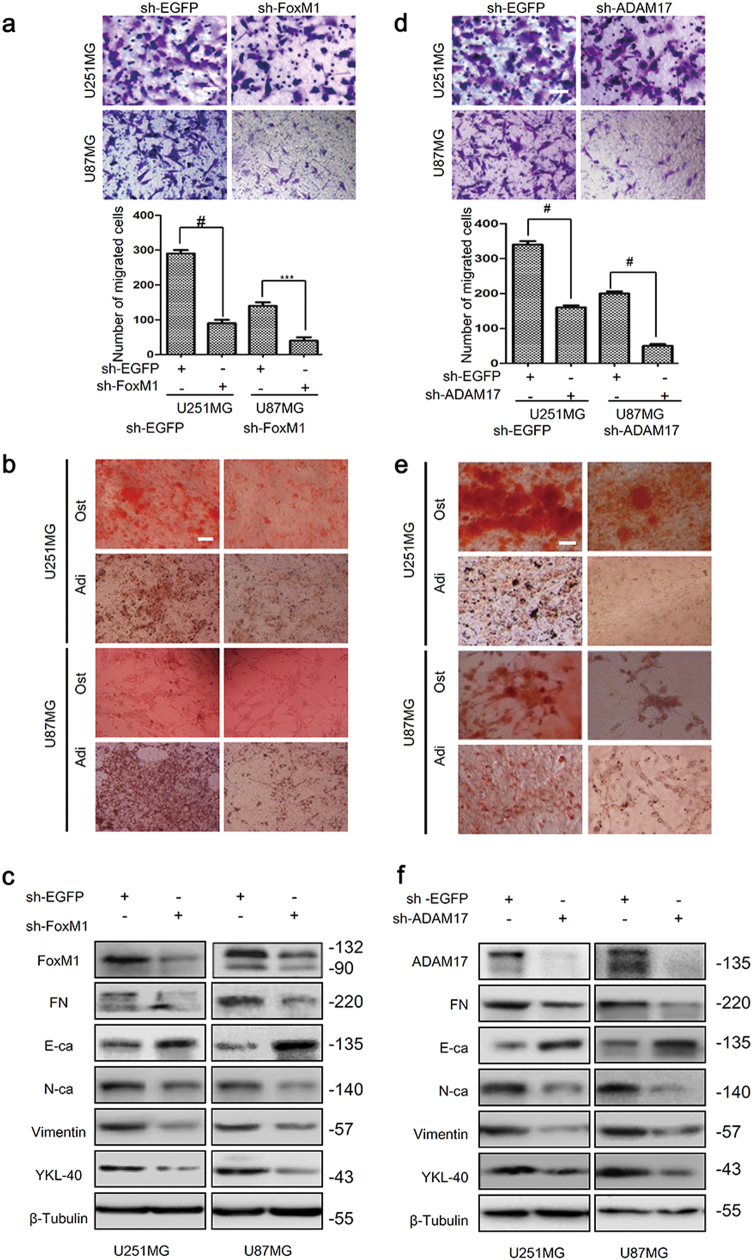
FoxM1 or ADAM17 knockdown inhibits mesenchymal transition in glioma cells. **a**, **b** FoxM1 knockdown reduced the migration as well as potentialities of adipogenesis and osteogenesis of U251MG and U87MG cells. Scale bar = 100 μm. The data are shown as the mean ± s.d. of three independent experiments. Student’s *t* test was used to determine the significance of the differences between the groups (***P < 0.001, ^#^*P* < 0.0001, Student’s *t* test). **c** The expression of mesenchymal markers and E-cadherin were detected after knocking down FoxM1 by western blot. **d**, **e** ADAM17 knockdown inhibited the migration, adipogenesis and osteogenesis of U251MG and U87MG cells. Scale bar = 100 μm. The data are shown as the mean ± s.d. of three independent experiments. Student’s *t* test was used to determine the significance of the differences between the groups (^#^*P* < 0.0001, Student’s *t* test). **f** The expression of mesenchymal markers and E-cadherin were detected after knocking down ADAM17 by western blot. The data are shown as the mean ± s.d. of three independent experiments. Student’s *t* test was used to determine the significance of the differences between the groups (***P < 0.001, ^#^*P* < 0.0001, Student’s *t* test)

To examine the roles of FoxM1 in MES transition of glioma cells, we first performed transfection using sh-FoxM1 and sh-EGFP plasmids in U251MG and U87MG cells. The transfection efficiency was examined by qRT-PCR and western blot (Supplementary Fig. S1a). Subsequently, we evaluated the mesenchymal phenotype of glioma cells after transfection. It was found that FoxM1 knockdown resulted in much lower abilities of migration, adipogenesis and osteogenesis compared with control groups (Fig. 2a, b). Furthermore, FoxM1 knockdown suppressed mesenchymal markers expression and promoted E-cadherin expression in U251MG and U87MG cells (Fig. 2c, Supplementary Fig. S3a, b). On the contrary, FoxM1 overexpression increased the abilities of migration, adipogenesis and osteogenesis, as well as the mesenchymal markers expression, while suppressed E-cadherin expression in SW1783 and LN229 cells (Supplementary Fig. S2b, S5a-c, S3c-d). Accordingly, it confirmed that FoxM1 was a critical regulator for MES transition in glioma cells.

### ADAM17 promotes MES transition in glioma cells

In order to verify the effects of ADAM17 on MES transition in glioma cells, we first knocked down ADAM17 in U251MG and U87MG cells (Supplementary Fig. S2c), and then investigated the biology behavior. The results showed that silencing ADAM17 weakened the abilities of migration, adipogenesis and osteogenesis in U251MG and U87MG cells (Fig. 2d, e). Furthermore, ADAM17 knockdown downregulated mesenchymal markers expression and elicited E-cadherin expression (Fig. 2f, Supplementary Fig. S3e–f), which is consistent with the results of FoxM1 knockdown in glioma cells. Subsequently, we found that ADAM17 overexpression obviously led to enhanced abilities of migration, adipogenesis, osteogenesis (Supplementary Fig. S2d, S5d–e) and contributed to the mesenchymal shift in SW1783 and LN229 cells (Supplementary Fig. S5f, S3g–h). Similarly, it was suggested that ADAM17 was also critical for the MES transition in glioma cells.

### ADAM17 is transcriptionally regulated by FoxM1 in glioma cells

**Fig. 3:**
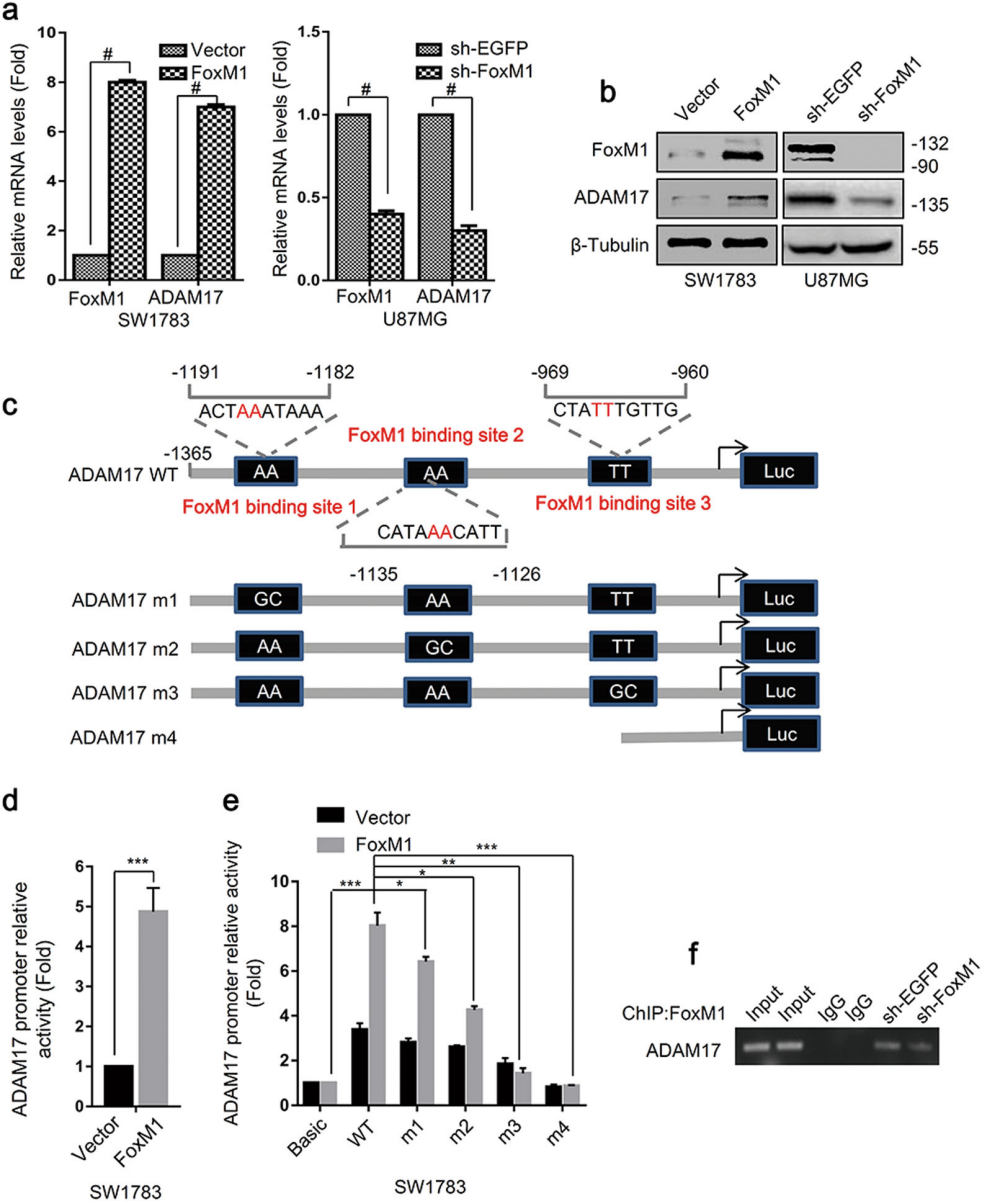
FoxM1 transcriptionally promotes ADAM17 expression through binding to the promoter of ADAM17. **a**, **b** qRT-PCR and western blot were performed to investigated the effects of altering the expression of FoxM1 on that of ADAM17 in glioma cells. The data are means ± SD from three independent experiments. Student’s *t* test was used to determine the significance of the differences between the groups (^#^*P* < 0.0001, Student’s *t* test). **c** The sites and mutant sites of FoxM1 binding to ADAM17 promoter. **d** FoxM1 overexpression increased the ADAM17 promotor activity. The data are shown as the mean ± s.d. of three independent experiments. Student’s *t* test was used to determine the significance of the differences between the groups (**P < 0.01, Student’s *t* test). **e** The luciferase report assay was used to determine the importance of the binding sites on ADAM17 promoter activity. The data are shown as the mean ± s.d. of three independent experiments. Student’s *t* test was used to determine the significance of the differences between the groups (*P < 0.05, **P < 0.01, ***P < 0.001, ^#^*P* < 0.0001, Student’s *t* test). **f** Results of ChIP assay performed with glioma cells to detect the binding between FoxM1 and ADAM17 promoter

The above results revealed that ADAM17 expression was highly associated with FoxM1 expression. Thus, we examined the effects of FoxM1 on ADAM17 mRNA and protein expression in glioma cells. FoxM1 overexpression dramatically increased ADAM17 mRNA level expression in SW1783 cells, whereas FoxM1 knockdown significantly reduced ADAM17 expression in U87MG cells (Fig.3a). Western blot analyses also confirmed the above results (Fig. 3b), suggesting that FoxM1 regulated ADAM17 expression. To investigate whether FoxM1 directly promotes the transcription of ADAM17, we identified 3 putative FoxM1-binding sites within ADAM17 promoter regions: 5′-ACTAAATAAA-3′, 5′-CATAAACATT-3′ and 5′-CTATTTGTTG-3′ located at −1365 to −960 bp upstream of the translation initiation site of ADAM17 (Fig. 3c). Subsequently, the ADAM17 promoter was cloned into pGL3-Basic vector, and then the promoter luciferase assay was conducted. The results revealed that overexpressing FoxM1 in SW1783 and HEK293T cells increased ADAM17 promoter activity (Fig. 3d, Supplementary Fig. S7a). Next, we generated four mutants of ADAM17 promoter, containing mutations in binding site 1, binding site 2 and binding site 3, and deletion mutant (Fig. 3c). We cotransfected FoxM1 combined with pGL3-ADAM17 wild-type (WT) or pGL3T-ADAM17 mutants into SW1783 and HEK293T cells. The results showed that the mutants exhibited decreased promoter activity compared with WT group, and deletion mutant exhibited the lowest transcriptional activity (Fig.3e, Supplementary Fig. S7b), indicating that all three FoxM1-binding sites were critical for ADAM17 promoter activity. In parallel, we also carried out ChIP analysis to study the occupancy of ADAM17 promoter by FoxM1. Consistent with the luciferase reporter assays, FoxM1 specifically interacted with these binding sites, and FoxM1 knockdown remarkably inhibited the binding of FoxM1 to this region (Fig. 3f). Taken together, these findings suggested that FoxM1 regulated ADAM17 expression via interacting with the FoxM1-binding sites at the ADAM17 promoter region.

### FoxM1/ADAM17 axis promotes MES transition in glioma cells

**Fig. 4:**
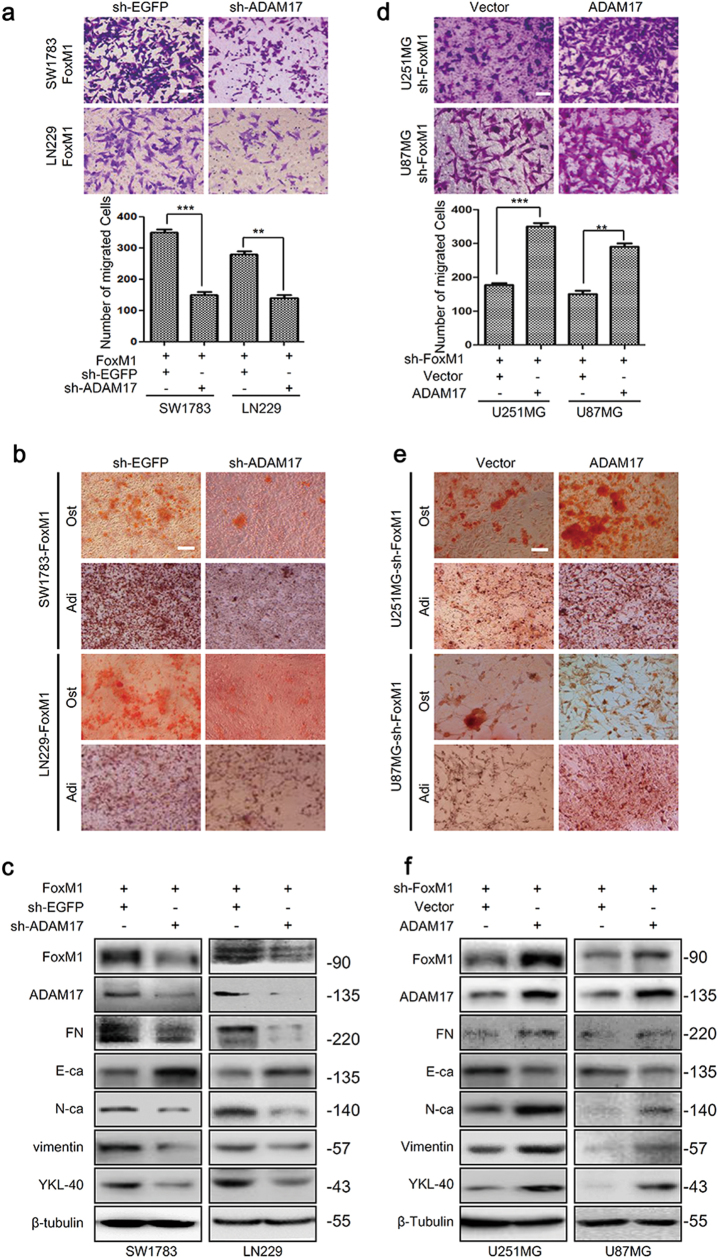
FoxM1/ADAM17 axis promotes mesenchymal transition in glioma cells. **a**, **b** SW1783 and LN229 cells were cotransfected with pCNA3.1-FoxM1 and sh-ADAM17 plasmids, and then determined the capacities of migration, adipogenesis and osteogenesis. Scale bar = 100 μm. The data are shown as the mean ± s.d. of three independent experiments. Student’s *t* test was used to determine the significance of the differences between the groups (**P < 0.01, ***P < 0.001, Student’s *t* test). **c** The effects of FoxM1 overexpression combined with ADAM17 knockdown on mesenchymal transition were investigated by western blot in SW1783 and LN229 cells. **d**, **e** The capacities of migration, adipogenesis and osteogenesis were determined after knocking down FoxM1 and overexpressing ADAM17 in U251MG and U87MG cells. Scale bar = 100 μm. Data are shown as the mean ± s.d. of three independent experiments. Student’s *t* test was used to determine the significance of the differences between the groups (**P < 0.01, ***P < 0.001, Student’s *t* test). **f** The effects of FoxM1 knockdown combined with ADAM17 overexpression on mesenchymal phenotype were investigated by western blot in U251MG and U87MG cells

To ascertain whether FoxM1 regulate MES transition in glioma cells in an ADAM17-dependedent manner, we first knocked down ADAM17 expression in FoxM1 overexpression cells. The results showed that the abilities of migration, adipogenesis and osteogenesis were strikingly inhibited (Fig. 4a, b), and the MES transition was blocked (Fig. 4c, Supplementary Fig. S3i–j). Similarly, ADAM17 overexpression in FoxM1 knockdown cells dramatically rescued the abilities of migration and potentialities of adipogenesis, osteogenesis (Fig. 4d, e) and induced the MES transition (Fig. 4f, Supplementary Fig. S3k–l). These data, taken together, were consistent with the hypothesis that FoxM1 promoted MES transition via ADAM17 in glioma cells.

### FoxM1/ADAM17 axis activates EGFR/AKT/GSK3β signaling and maintains FoxM1 stability in glioma cells

**Fig. 5:**
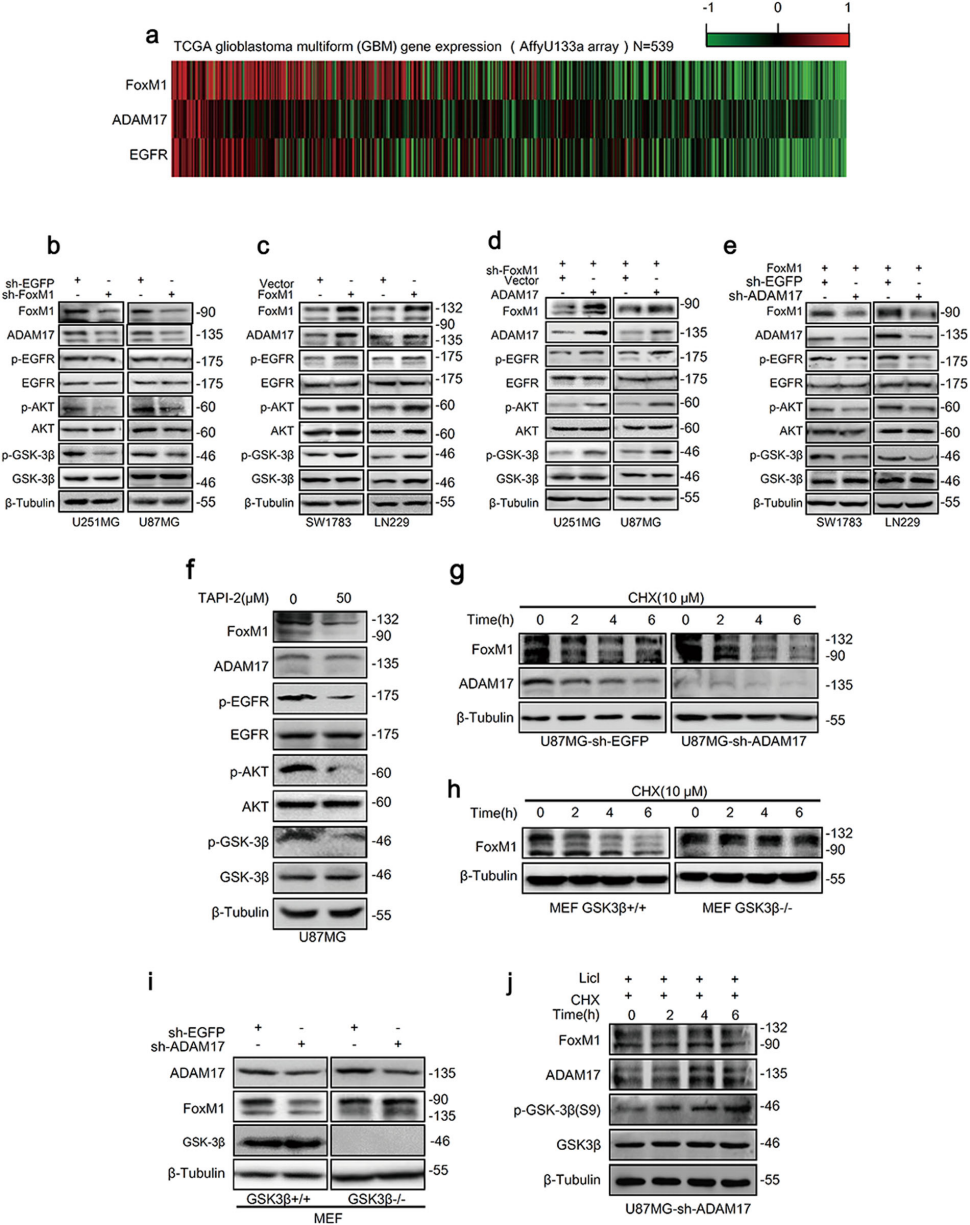
FoxM1/ADAM17 axis drives EGFR/AKT/GSK3β signaling and maintains FoxM1 stability in glioma cells. **a** Heatmap revealed that the expression of FoxM1 and ADAM17 were correlated with that of EGFR in glioblastoma samples from TCGA database. **b**, **c** The effects of FoxM1 on EGFR/AKT/GSK3β signaling pathway in glioma cells were investigated by western blot. **d** The key components of EGFR/AKT/GSK3β signaling were detected after knocking down FoxM1 and overexpressing ADAM17 by western blot in U251MG and U87MG cells. **e** The effects of FoxM1 upregulation and ADAM17 knockdown on EGFR/AKT/GSK3β signaling pathway were confirmed by western blot in SW1783 and LN229 cells. **f** U87MG cells were treated with TAPI-2(ADAM17 inhibitor), and then indicated proteins were measured by western blot. **g** After transfected with sh-ADAM17, U87MG cells were treated with CHX (0, 2, 4, 6 h) and then western blot was used to determine the expression of FoxM1. **h** FoxM1 levels were measured in MEF GSK3β+/+ and GSK3β−/− cells with CHX (0, 2, 4, 6 h) . **i** The effects of ADAM17 downregulation on FoxM1 expression in MEF GSK3β+/+ and GSK3β−/− cells. **j** The levels of indicated proteins were detected by western blot in U87MG-sh-ADAM17 cells treated with CHX and LiCl (0, 2, 4, 6 h)

ADAM17 was related to the activation of the EGFR as previously described^34^. Thus, to explore the mechanisms underlying the effects of FoxM1/ADAM17 axis on MES transition, we first examined the expression of FoxM1, ADAM17 and EGFR in GBM using the data from TCGA. We found that EGFR expression was correlated with FoxM1 and ADAM17 expression in patients (Fig. 5a, Supplementary Fig. S1a). Next, the key components of EGFR/AKT/GSK3β signaling pathway were examined. The results turned out that FoxM1 knockdown expectedly reduced the phosphorylation of EGFR, AKT and GSK3β in U87MG, and U251MG cells (Fig.5b, Supplementary Fig. S4a-b), while FoxM1 overexpression activated the EGFR/AKT/GSK3β pathway in SW1783 and LN229 cells (Fig. 5c, Supplementary Fig. S4c–d). Furthermore, exogenous ADAM17 was able to rescue the inactivation of the EGFR/AKT/GSK3β pathway because of FoxM1 knockdown in U251MG and U87MG cells (Fig. 5d, Supplementary Fig. S4e–f), whereas ADAM17 knockdown could abolish the activation of the signaling pathway due to FoxM1 overexpression in SW1783 and LN229 cells (Fig. 5e, Supplementary Fig. S4g–h), indicating that FoxM1/ADAM17 axis activated EGFR/AKT/GSK3β signaling.

Of note, FoxM1 expression was increased or reduced following ADAM17 expression alteration (Fig. 5d, e), hinting that ADAM17 expression might regulate FoxM1 expression. Taken into consideration that ADAM17 expression alteration had no effects on the mRNA levels of FoxM1 (Supplementary Fig. S6), and ADAM17 is one of the major ectodomain shedding proteinases^35^, we treated U87MG cells with ADAM17 inhibitor TAPI-2 (50 μM). As expected, FoxM1 protein level was reduced compared to control group, as well as the phosphorylation of EGFR, AKT and GSK3β (Fig. 5f, Supplementary Fig. S4i), implicating that ADAM17 regulated FoxM1 expression in protein levels. We further sought to confirm that ADAM17 regulated FoxM1 expression by controlling its degradation. Treating U87MG-sh-EGFP and U87MG-sh-ADAM17 cells with CHX for 0, 2, 4 and 6 h, the protein levels of FoxM1 were both reduced gradually, but much faster in U87MG-sh-ADAM17 group (Fig. 5g, Supplementary Fig. S4j). Furthermore, to explore whether GSK3β mediate FoxM1 degradation, we treated MEFGSK3β+/+ and MEF GSK3β−/− cells with CHX (10 μM) for 0, 2, 4 and 6 h, as shown in Fig. 5h, FoxM1 expression was decreased in time-dependent manner in MEF GSK3β+/+ cells, while almost constant in GSK3β−/− group (Supplementary Fig. S4k). Knocking down ADAM17 levels reduced the expression of FoxM1 in MEF GSK3β+/+ cells, while it had no effects in MEF GSK3β−/− cells (Fig. 5i, Supplementary Fig. S4l). Treating U87MG-sh-ADAM17 with CHX for 0, 2, 4 and 6 h, FoxM1 was reduced. However, at the same time, when treated with LiCl (GSK3β inhibitor), the phosphorylation of GSK3β (S9) was increasing gradually, and the expression of FoxM1 was not altered (Fig. 5j, Supplementary Fig. S4m), showing that GSK3β was an important regulator of FoxM1 stability. These data suggested that ADAM17/EGFR/GSK3β pathway mantained the highly expression of FoxM1 in glioma cells.

### FoxM1/ADAM17 axis promotes tumor malignancy in vivo

**Fig. 6:**
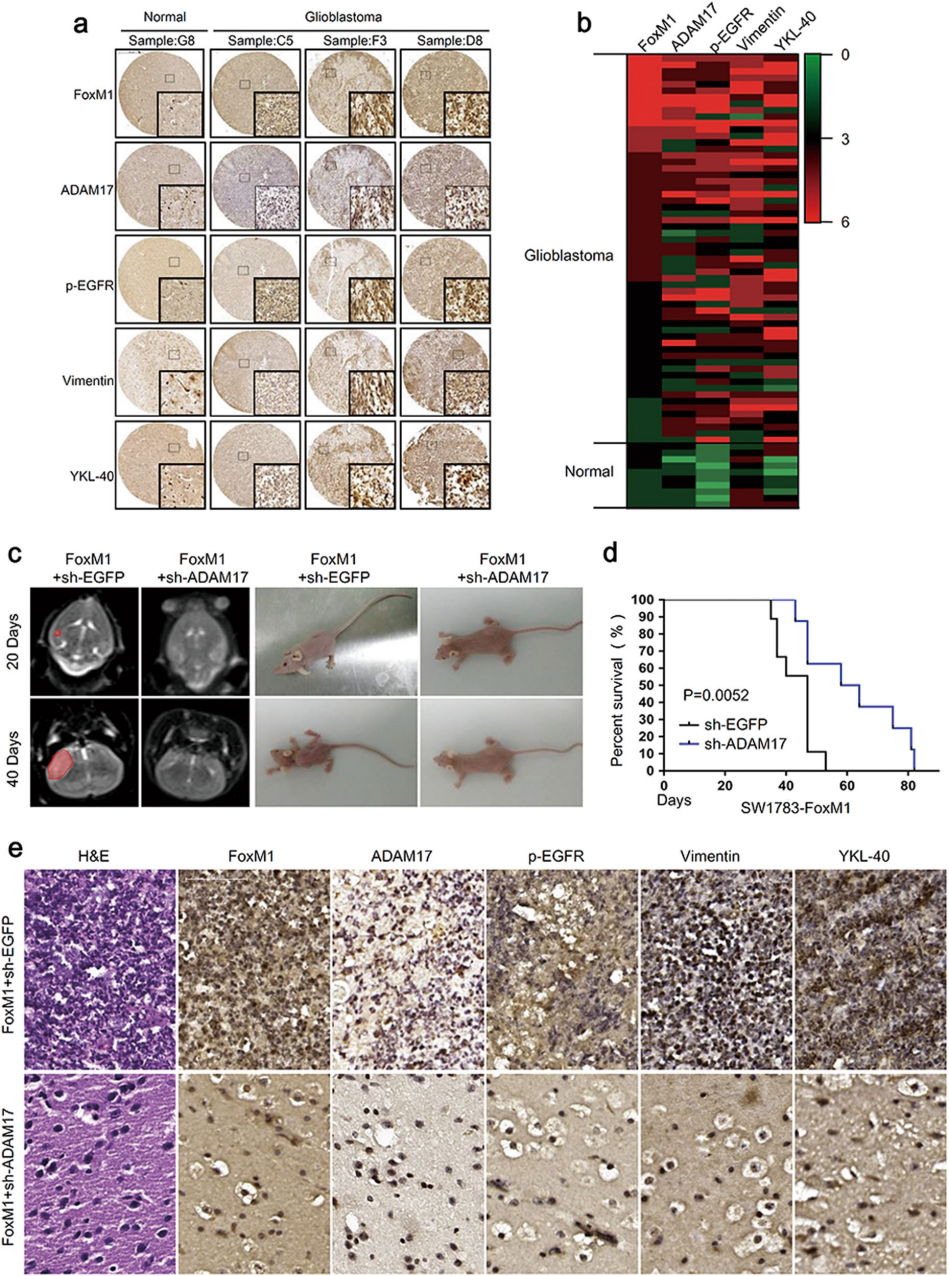
FoxM1/ADAM17 axis promotes the tumor progression in vivo. **a** IHC analyses were used to determine the expression of FoxM1, ADAM17, p-EGFR and mesenchymal markers expression in human glioblastoma samples. Scale bar = 500 μm **b** The heatmap revealed that ADAM17 expression was highly associated with FoxM1 expression and both of them were correlated with mesenchymal phenotype in GBM. **c** Twenty and 40 days after tumor implantation, mice were fixed in mice MRI equipment to evaluate the volume of xenograft tumors. **d** Kaplan–Meier algorithm was performed for evaluating overall survival time of mice between FoxM1-sh-EGFP and FoxM1-sh-ADAM17 groups. *P < 0.05. **e** HE staining and immunochemistry assays were used to assess the promoting effects of FoxM1/ADAM17 axis on tumor malignancy. Scale bar = 500 μm

To confirm the clinical relevance of FoxM1/ADAM17 axis in GBM, we examined the protein levels of FoxM1, ADAM17 and mesenchymal markers in human primary GBM samples by tissue microarray (Fig. 6a). Gene expression heat maps revealed that ADAM17 expression was highly associated with FoxM1 expression in patients and both of them were correlated with mesenchymal phenotype in GBM (Fig. 6b). For further investigation, quantification of staining and linear regression analyses were performed to show a significant correlation between FoxM1 and ADAM17 expression. On the other hand, the expression of ADAM17 was statistically significant to those of FoxM1, p-EGFR and YKL-40 (Supplementary Fig. S7a–b). These results further supported a critical role of the FoxM1/ADAM17/EGFR pathway in promoting tumor progression of human glioblastoma, implicating that FoxM1/ADAM17 axis might involve in tumorigenicity of glioma cells in vivo.

We next confirmed whether the FoxM1/ADAM17 axis regulated the tumorigenicity of glioma cells. It has been reported that exogenous FoxM1 significantly enhances tumor growth^36^. In the current study, ADAM17 knockdown in SW1783-FoxM1 cells dramatically impeded the tumor growth and increased the biologic status and survival of mice (Fig. 6c, d). As a brain tumor grew, it may interfere with the normal functions of the brain of mice. Since the skull cannot expand in response to the growth of a tumor, the first symptoms were usually due to increased pressure in the brain. Headaches, seizures, and changes in behavior are the most common symptoms. As a result, the mice lost weight and their heads were kept to one side called the compulsive position showed in the right part of Fig. 6c. The mice in FoxM1 + sh-EGFP group were thinner than those in FoxM1 + sh-ADAM17 group 20 days after implanting the tumor. Forty days later, the mice became much thinner. We further analyzed FoxM1, ADAM17, p-EGFR and mesenchymal markers expression in brain tissues obtained from the xenograft mice. FoxM1 overexpression combined with ADAM17 knockdown exhibited low levels of p-EGFR and mesenchymal markers compared to FoxM1 overexpression without ADAM17 knockdown in SW1783 cells (Fig. 6e). These data demonstrated that ADM17 was critical for FoxM1 promoting the tumorigenic potential of glioma cells, and FoxM1/ADAM17 axis promoted tumor progress in vivo.

## Discussion

Herein, we clarified that FoxM1 and ADAM17 were co-expressed in GBM, and FoxM1/ADAM17 axis induced MES transition via EGFR/AKT/ GSK3β signaling, which in turn led to the elevated expression of FoxM1. Together, the FoxM1/ADAM17 feedback loop is essential for the malignant phenotype of GBM.

GBM is the most aggressive form of brain cancer due to its highly invasive nature, and MES transition has been pointed as one of the mechanisms that confer to GBM cells this invasive property^37^. To date, abundant experimental evidences have firmly explained that FoxM1 played important roles in GSC self-renew, metastasis, angiogenesis, radio-resistance, temozolomide (TMZ) resistance, and tumorigenesis^24,36,38^. Whereas ADAM17 played similar roles in inducing malignant phenotype of glioma cells, mostly restricting to shedding of ErbB ligands such as activating EGFR signaling pathway^39–42^. However, whether they were involved in MES transition remained unclear and the crosstalk of them has not yet been discussed. Here we demonstrated that the expression profiles of FoxM1 and ADAM17 were both significantly correlated with the mesenchymal phenotype and overall patient survival of GBM. Notably, it was the first time to demonstrate that there was a direct link between FoxM1 and ADAM17: FoxM1 binding to the promotor of ADAM17 to regulate its expression, which even elucidated a mechanism for overexpression of ADAM17 in glioma. Furthermore, our research is the first to explain the FoxM1/ADAM17 axis in promoting MES transition of GBM.

EGFR is usually overexpressed in glioma cells^43^, and its downstream signaling cascades are frequently activated during the development of malignancy. EGFR can be activated by ectodomain release of EGFR ligands, including epiregulin, TGF-α, amphiregulin, and heparin-binding EGF-like growth factor, which were mediated by ADAM17^44^. EGFR ligands binding resulted in receptor self-dimerization, auto-phosphorylation and subsequent activation of downstream PI3K/AKT and Ras/MAPK pathways, mediating transcription of genes required for the growth and progression of tumor^45–47^. Moreover, FoxM1 was able to activate AKT/GSK3β/Snail pathway^18^, which implicated crosstalk existence. In this study, the expression levels of the coexpressed genes (FoxM1, ADAM17 and EGFR) were proved varied in a continuous and coordinate manner across the samples in TCGA. In addition, FoxM1 activated EGFR/AKT/GSK3β signaling pathway in glioma cells. Collectively, it suggests that ADAM17/EGFR signaling pathway is driven by FoxM1, which promotes the MES transition and contributed in the progress of GBM.

Intriguingly, ADAM17 affected the stability of FoxM1 via activating GSK3β. A recent study showed that Wnt signaling activation inhibited the GSK3β-induced FoxM1 phosphorylation and led to interaction between FoxM1 and deubiquitinating enzyme USP5, thereby deubiquitination and stabilization of FoxM1^20^. Here we elaborated that high level of ADAM17 could also triggered the phosphorylation of GSK3β on Ser9, and then its inactivation, which led to deubiquitination and stabilization of FoxM1. These results demonstrate that ADAM17 regulates the stability of FoxM1 via EGFR/AKT/GSK3β signaling pathway and maintains the high expression of FoxM1 in GBM.

In summary, we emphasize that FoxM1 drives ADAM17/EGFR signaling pathway, and then stabilizes its expression, and in turn promotes the MES transition in GBM. These findings further suggest that this activation loop could be a potential target for novel therapeutic strategies against malignant glioma.

## Materials and methods

### Gene expression omnibus profile and TCGA

Gene expression data was downloaded from The Cancer Genome Atlas data through the Cancer Brower site (https://genome-cancer.ucsc.edu/proj/site/hgHeatmap/), and analyzed for GBM.

### Cell lines and cell culture

Human glioma cell lines, SW1783, LN229, U87MG and U251MG were obtained from ATCC (Manassas, VA, USA), cultured at 37 ℃ in a 5% CO_2_ atmosphere and maintained with Dulbecco’s Modified Eagle’s Medium (DMEM) containing 10% fetal bovine serum (FBS) within 3 months of resuscitation from the frozen stock, with fewer than 20 passages. DMEM, FBS and trypsin were purchased from Gibco. All the cell lines were authenticated by short-tandem-repeat (STR) profiling.

### Quantitative PCR analysis

Total RNA was extracted using RNAiso Plus (Takara). Reverse transcription was performed using RevertAid First Strand cDNA Synthesis Kit (Thermo) according to the manufacturer’s specification. QRT-PCR was performed in triplicate in 20 μl reactions with iQ SYBR Premix Ex Taq Perfect Real Time (Bio-Rad Laboratories, Inc.). The relative mRNA content was calculated using the 2^−ΔΔCT^ method with GAPDH as an endogenous control. The sequences of primers used for PCR are described in Supplementary Table 1. All experiments were performed in biological triplicate.

### Western blot analysis

The cultured cells were rinsed with cold PBS before treated with RIPA lysis buffer at 4 °C for 10 min. Then the mixture was heated at 100 °C for 10 min and centrifuged under 4 °C at 12000 × *g* min^−1^ for 10 min. About 20 μg of protein was loaded in each lane, separated by 10% SDS-PAGE and transferred to the PVDF membrane. The membrane was blocked with 5% BSA for 1 h at room temperature before overnight incubation with primary antibodies 4 °C, followed by the secondary antibody. All primary antibodies were used at the dilution according to manufacturer: anti-FoxM1 (Santa Cruz, sc-500), anti-fibronectin (Santa Cruz, sc-8422), anti-ADAM17 (CST, 3976), anti-vimentin (CST, 5741), anti-E-cadherin(CST, 3195), anti-N-cadherin(CST, 13116), anti-p-EGFR (Tyr1068) (CST, 3777), anti-EGFR (CST, 2085), anti-p-AKT (S473) (CST, 4060), anti-AKT (CST, 9272), anti-GSK3β (CST, 12456), and anti-p-GSK3β (Ser9) (CST, 9323), YKL-40 (BioWorld, BS6564) and anti-β-tubulin (Thermo, MA5-11732). Bound antibodies were detected using goat anti-rabbit (Santa Cruz, sc-2004) and goat anti-mouse secondary antibodies (Santa Cruz, sc-2005) conjugated with horseradish peroxidase and visualized using enhanced chemiluminescence substrate (RJT Biosystems, 17001). β-Tubulin was used as a loading control. All experiments were performed in biological triplicate.

### Immunofluorescent

A total of 1 × 10^4^ cells were plated on coverslips and incubated at 37 °C for 48 h. Cells were fixed with 4% paraformaldehyde for 20 min, permeabilized in 0.3% Triton X-100 for 10 min and washed 3 times with PBST (0.05% Tween-20 in PBS). After blocking in 3% bovine serum albumin, the coverslips were incubated with primary antibodies overnight at 4 °C: anti-FoxM1 (Santa Cruz, sc-500), anti-ADAM17 (CST, 3976), anti-vimentin (CST, 5741) and YKL-40 (BioWorld, BS6564). Staining was achieved with the secondary antibodies for 1 h, and Hoechst 33342 (1 μg/ml; Sigma-Aldrich) was used to counterstain the nuclei for 5–10 min. Cover the coverslips with glycerin and view under a fluorescence microscope (Leica). Each test was repeated in triplicate.

### Transient transfection

Cells were seeded to be 70–90% confluent at transfection in six-well plates. The following day, transfection was done according to the protocol, using the Lipofectamine® 2000 transfection reagent (Invitrogen). After incubation for another 48 h, the cells were determined using qRT-PCR and western blot analysis. Each test was repeated in triplicate.

### Promoter reporters and dual luciferase assay

The ADAM17 promoter was cloned into pGL3-Basic vector at XhoI and HindIII sites. The ADAM17 mutant promoter constructs at the putative FoxM1-binding sites were generated using the QuikChange® XL Site-Directed Mutagenesis Kit (Agilent Technologies, 200516). Sequences of promotor and mutant promotors used are described in Supplementary Table 2. For dual-luciferase assays, the cells were cultured for 48 h after transfection, and cell lysate was prepared to measure luciferase activity using the dual luciferase reporter assay system (Promega, E1910) according to the protocol of manufacturer. All experiments were conducted in triplicate.

### Chromatin immunoprecipitation assays

Chromatin immunoprecipitation (ChIP) assays were performed using the ChIP assay kit (Cell Signaling Technology) according to the manufacturer’s protocol. Briefly, 1 × 10^7^ cells were cross-linked with 1% formaldehyde for 10 min at 37 °C. Subsequently, chromatin was digested into fragments of 150–900 bp by micrococcal nuclease (400 gel units) for 20 min at 37 °C, followed by ultrasonic disruption of the nuclear membrane using a standard microtip and Branson W250D Sonifier (four pulses, 60% amplitude, duty cycle 40%). For immunoprecipitation, total chromatin was incubated overnight at 4 °C with 5 μg of the respective antibodies and rabbit IgG served as a negative control. The resulting precipitated DNA samples were analyzed by PCR using primers to amplify a potential binding site region of the ADAM17 promoter with specific primers. PCR products were resolved electrophoretically in a 2% agarose gel and visualized by ethidium bromide staining. The sequences of primers used for PCR are described in Supplementary Table 1. Each test was repeated in triplicate.

### Transwell migration assay

Transwell migration assays was carried out using transwell inserts (Corning) containing 8 μm permeable pores according to the manufacturer’s protocol. Transfected cells were collected, resuspended in serum-free medium and transferred to 8 μm permeable pores (1 × 10^4^ cells per well). The chambers were then incubated for 10 h in culture medium with 10% FBS in the bottom chambers before examination. The cells on the upper surface were scraped and washed away, whereas the migrated cells on the lower surface were fixed and stained with 0.05% crystal violet for 30 min. Finally, five independent fields were counted for each transwell and the average number of cells per field was represented in the graphs. Each test was repeated in triplicate.

### Osteogenesis assay

Cells were cultured in 24-well plates, medium for osteogenesis induction were 10% FBS, 1 μM dexamethasone, 10 μM β**-**sodium glycerol-phosphate and 50 mg/L vitamin C in DMEM. The medium was changed every 2 days for about 21 days. Cells were fixed with 4% paraformaldehyde for 20 min, and stained with alizarin red for 30 min. Each test was repeated in triplicate.

### Adipogenesis assay

Cells were cultured in 24-well plates, medium for adipogenesis induction were 10% FBS, 2 μM insulin, 500 μM IBMX, 1 μM dexamethasone and 200 μM indomethacin in DMEM; Medium for adipogenesis maintenance were 10% FBS and 2 μM insulin in DMEM. Cells were cultured with adipogenesis induction medium for 2 days and adipogenesis maintenance medium for 1 day. 3 cycles later, the cells were fixed with 4% paraformaldehyde for 20 min, and stained with oil red for 30 min. Each test was repeated in triplicate.

### Immunohistochemistry

Tissue sections were air-dried, deparaffinized and rehydrated. Endogenous peroxidase activity was blocked for 10 min with H_2_O_2_ (3%). Antigen retrieval was done with citrate buffer in a pressure cooker for 10 min. 5% serum-free protein block from Biosharp was used for 20 min for blocking non-specific antibody binding. Slides were then incubated overnight at 4 °C with primary antibody and at room temperature for 30 min with species-specific secondary antibody (BOSTER, SA1020). For negative controls, the primary antibody was omitted and replaced by negative IgG. The reaction was developed with DAB (BOSTER, AR1022) and slides were counterstained with hematoxylin. Immunostaining intensity and reactivity were examined by CaseViewerafter scanning using digital microscope application under a ×40 magnification objective. Protein expression was quantified using a 4-value intensity score (0, none; 1, weak; 2, moderate; and 3, strong) and4-value percentage score (0, <10%; 1, 10–40%; 2, 40–70%; 3, >70%). A final expression score was obtained by using the intensity values plus reactivity extension values. Staining was evaluated blindly by 3 investigators.

Tissue Microarray was purchased from Easy Biotrade. Human glioma samples used in the present study were confirmed by pathologist according to WHO criteria. The use of human specimens was approved by the relevant institutional review boards.

### Intracranial tumor assay

Experiments were performed in accordance with the National Institutes of Health guide for the care and use of Laboratory animals (NIH Publications No. 8023, revised 1978). All mouse experiments were approved by the Institutional Animal Committee of Jiangsu University. Six- to eight-week-old female athymic BALB/c nude mice at an average weight of 20 g were purchased from SLRC Laboratory Anima. Mice were departed into two groups randomly and blindingly. In this study, all the surgical procedures were carried out under general anesthesia by intraperitoneal injection of 1% Pentobarbital Sodium (50 mg/kg). A small burr hole, 2 mm in diameter was made 2 mm to the midline and 0.5 mm anterior to bregma using micro skull drill. Micromanipulator packed with glioma cells was navigated to a depth of 2 mm via skull hole. A total of 5 × 10^5^ cells in 10 μl of serum-free DMEM medium were slowly and smoothly injected into the caudate/putamen nuclei of the mouse brain within 1 min. Skull hole was sealed with bone wax and scalp sutured. The weight and survival time of each mouse were monitored. Nine mice for SW1783FoxM1-sh-EGFP group and eight mice for SW1783FoxM1-sh-ADAM17 group were done. Mice were examined on day 20 and day 40 post tumor implantation to detect the growth of the grafted tumor cells. After anesthetized as the same way described above, mice were fixed in mice MRI equipment. A3T MAGNETOM-Trio-Tim (Siemens AG) was used for brain imaging. Scanning parameters was as follows: layer thickness: 1.5 mm; space between layers: 0.15 mm T2WI: TR3500 ms and TE90 ms. Animals showing general or local symptoms were killed, and brains were extracted and fixed in 4% paraformaldehyde for 24 h, embedded in paraffin, and sectioned into 5 mm slices for H&E and immunohistochemistry.

### Statistical analysis

Statistical analyses were performed using GraphPad Prism 5 (GraphPad). The statistical analyses were performed by Student’s *t* test between two groups and one-way ANOVA for more than two groups comparison. Survival curves were determined using the Kaplan–Meier method and differences between groups were estimated by the Log-rank test. The variance between the groups that are being statistically compared is similar. The data are shown as the mean ± s.d. of three independent experiments. Statistical significant: *P < 0.05, **P < 0.01, ***P < 0.001, ^#^*P* < 0.0001.

## Electronic supplementary material

Figure S1

Figure S2

Figure S3

Figure S4

Figure S5

Figure S6

Figure S7

Figure S8

Supplementary figure legends -CDDis-revised

Supplementary tables
