# Supplementary figures and images for: FoxM1 drives ADAM17/EGFR activation loop to promote mesenchymal transition in glioblastoma
Source: Cell Death Dis. 2018 Apr 27;9(5):469. doi: 10.1038/s41419-018-0482-4 (PMC5920065; doi:10.1038/s41419-018-0482-4)

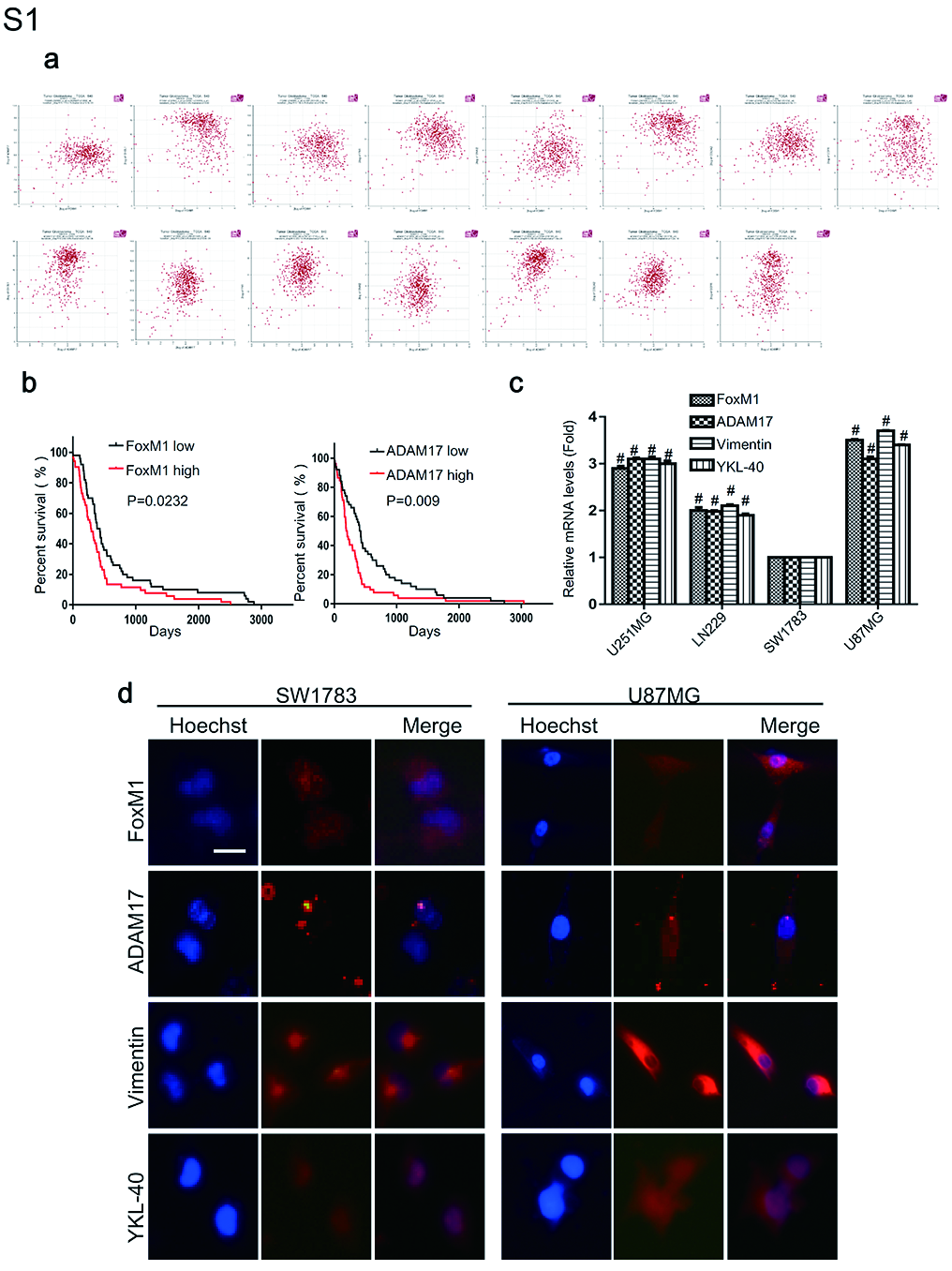

Supplement: Supplementary file 1 — Figure S1 [file 41419_2018_482_MOESM1_ESM.tif]

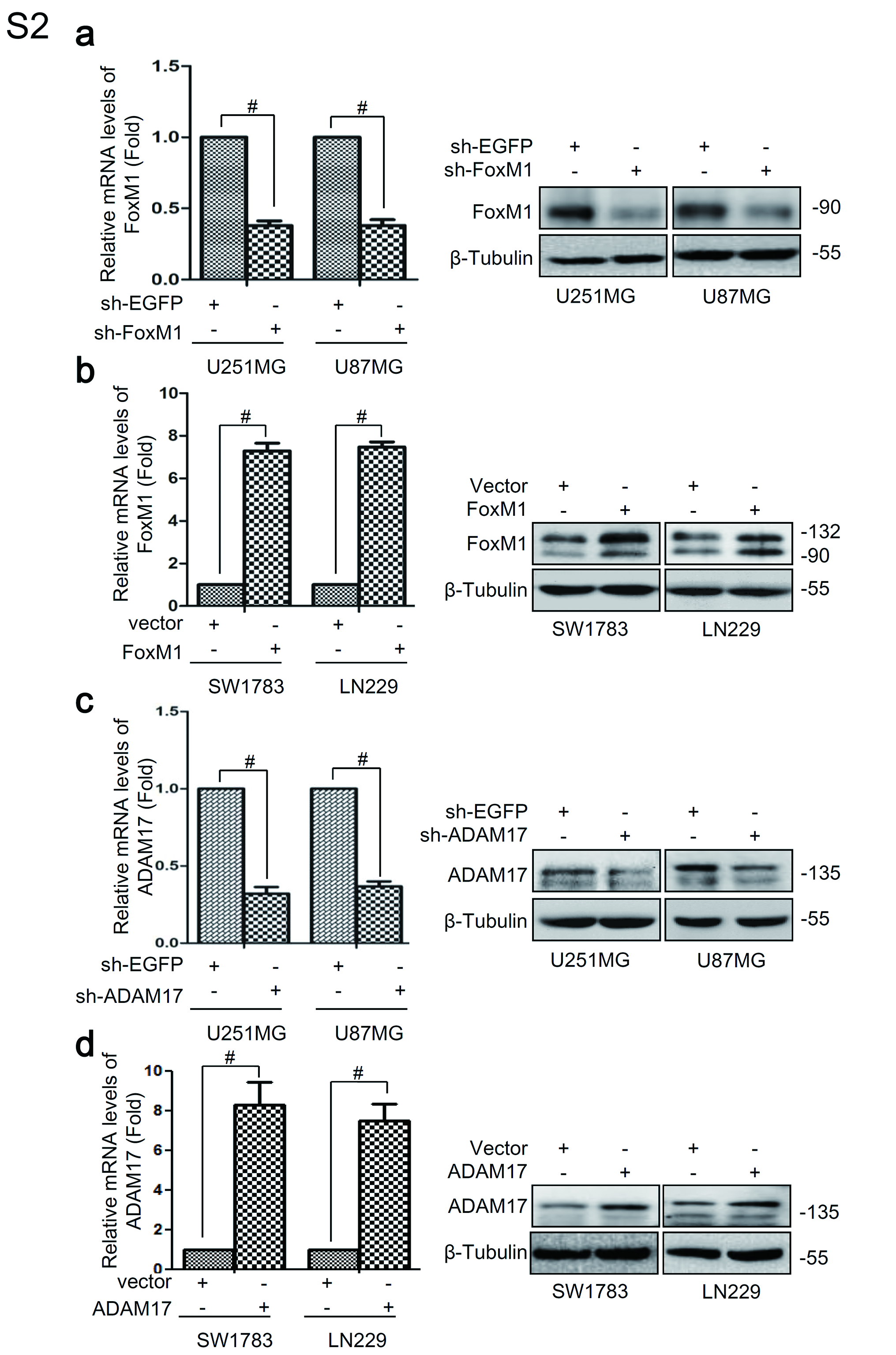

Supplement: Supplementary file 2 — Figure S2 [file 41419_2018_482_MOESM2_ESM.tif]

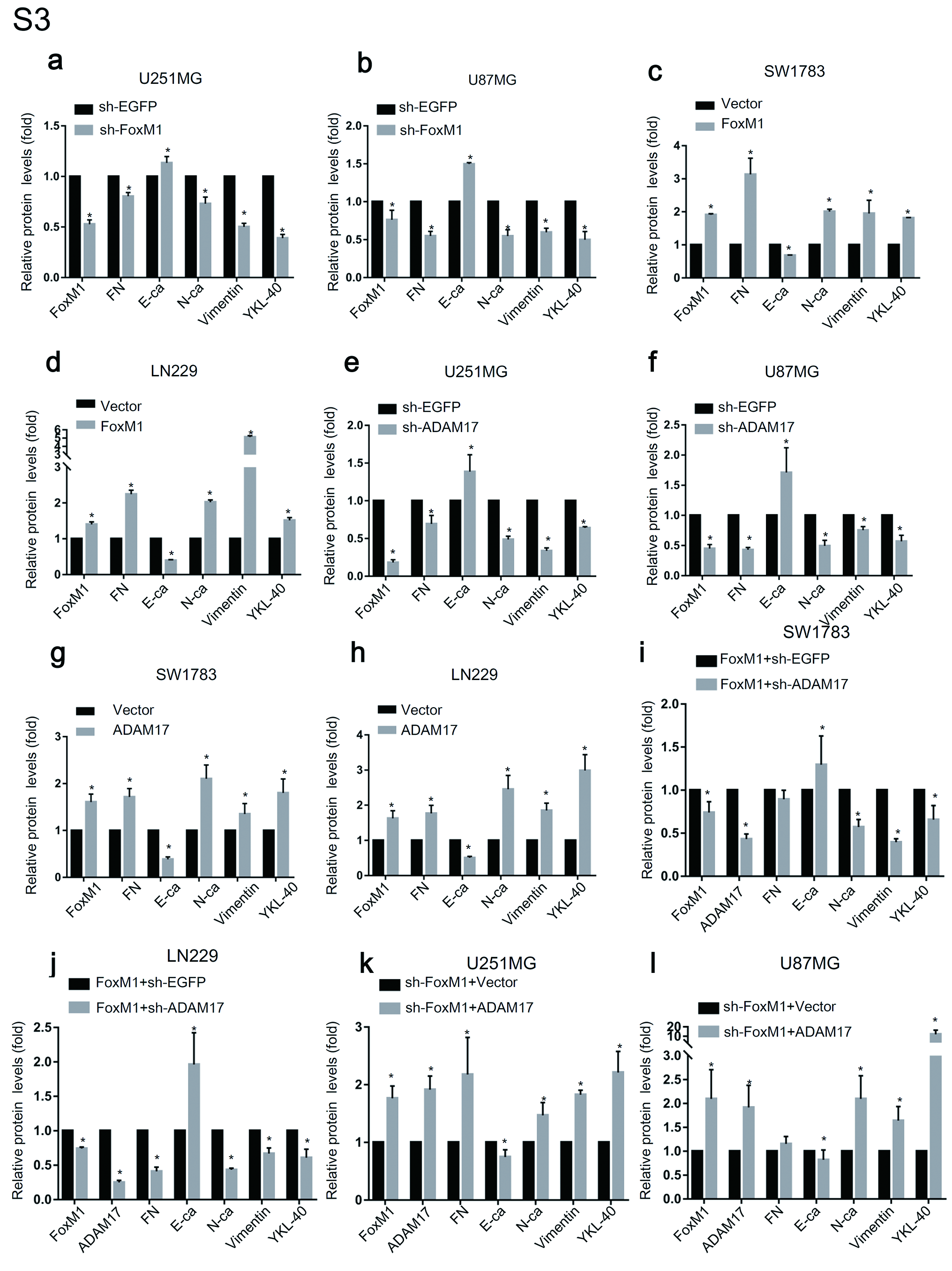

Supplement: Supplementary file 3 — Figure S3 [file 41419_2018_482_MOESM3_ESM.tif]

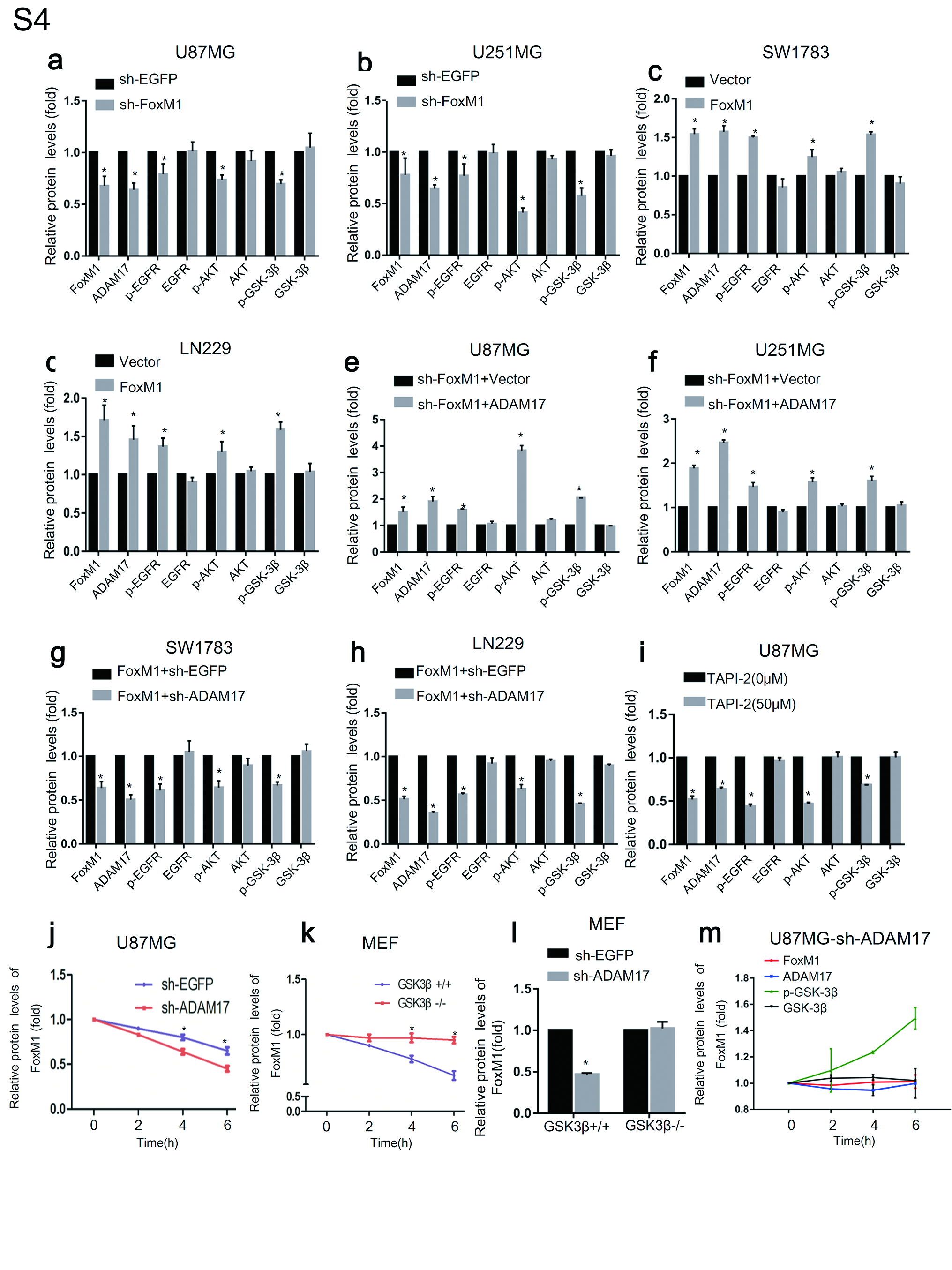

Supplement: Supplementary file 4 — Figure S4 [file 41419_2018_482_MOESM4_ESM.tif]

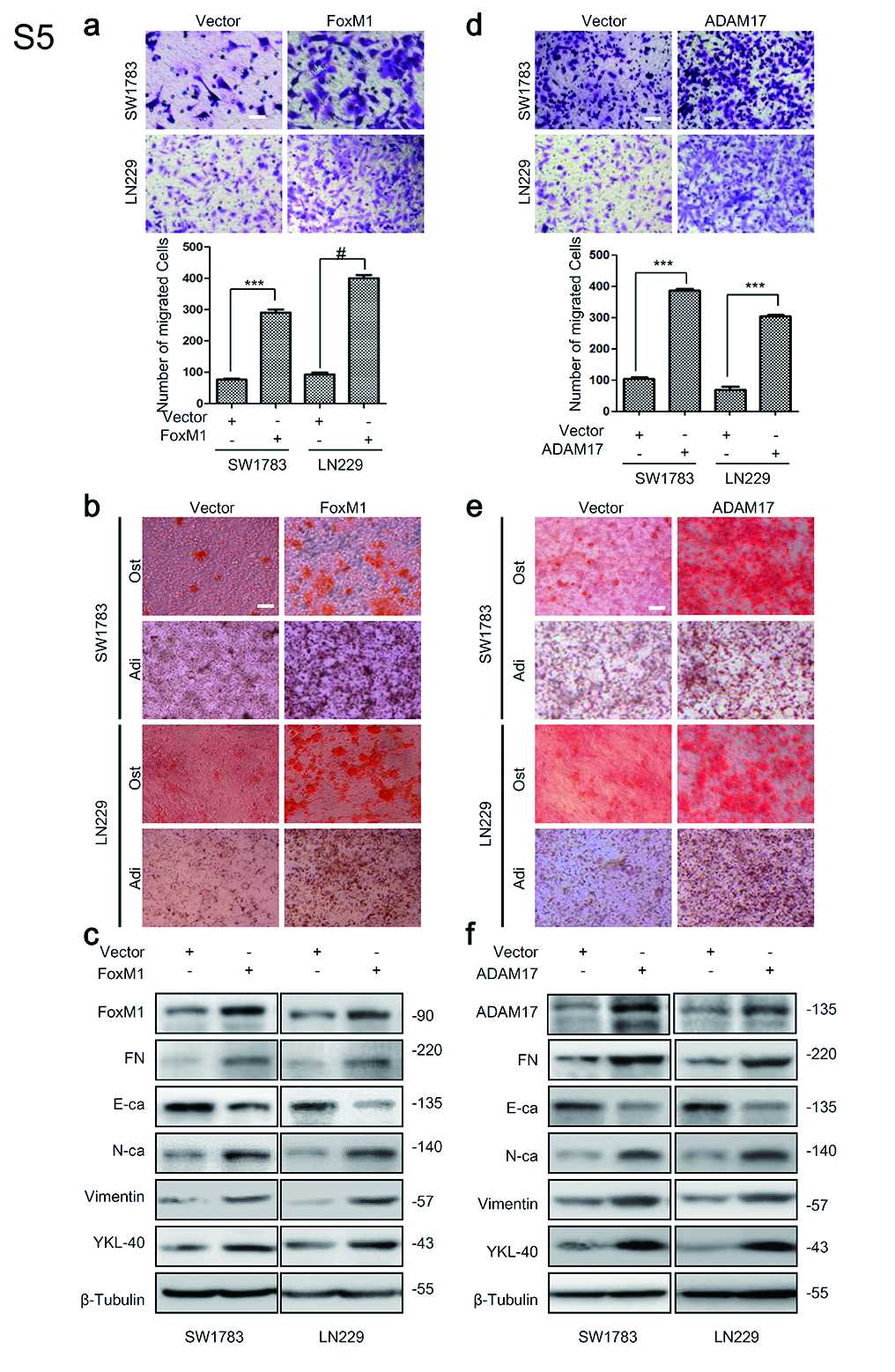

Supplement: Supplementary file 5 — Figure S5 [file 41419_2018_482_MOESM5_ESM.tif]

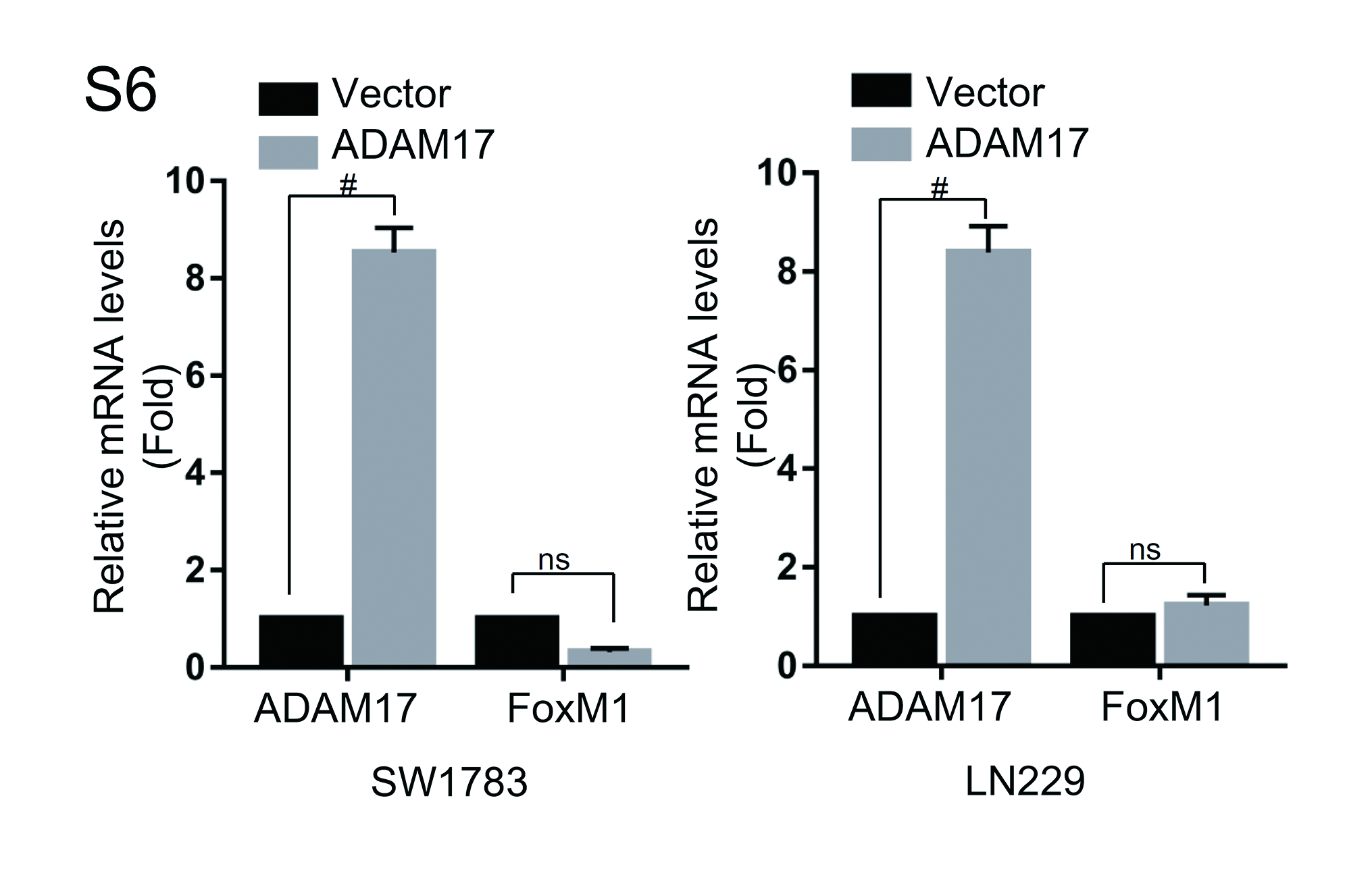

Supplement: Supplementary file 6 — Figure S6 [file 41419_2018_482_MOESM6_ESM.tif]

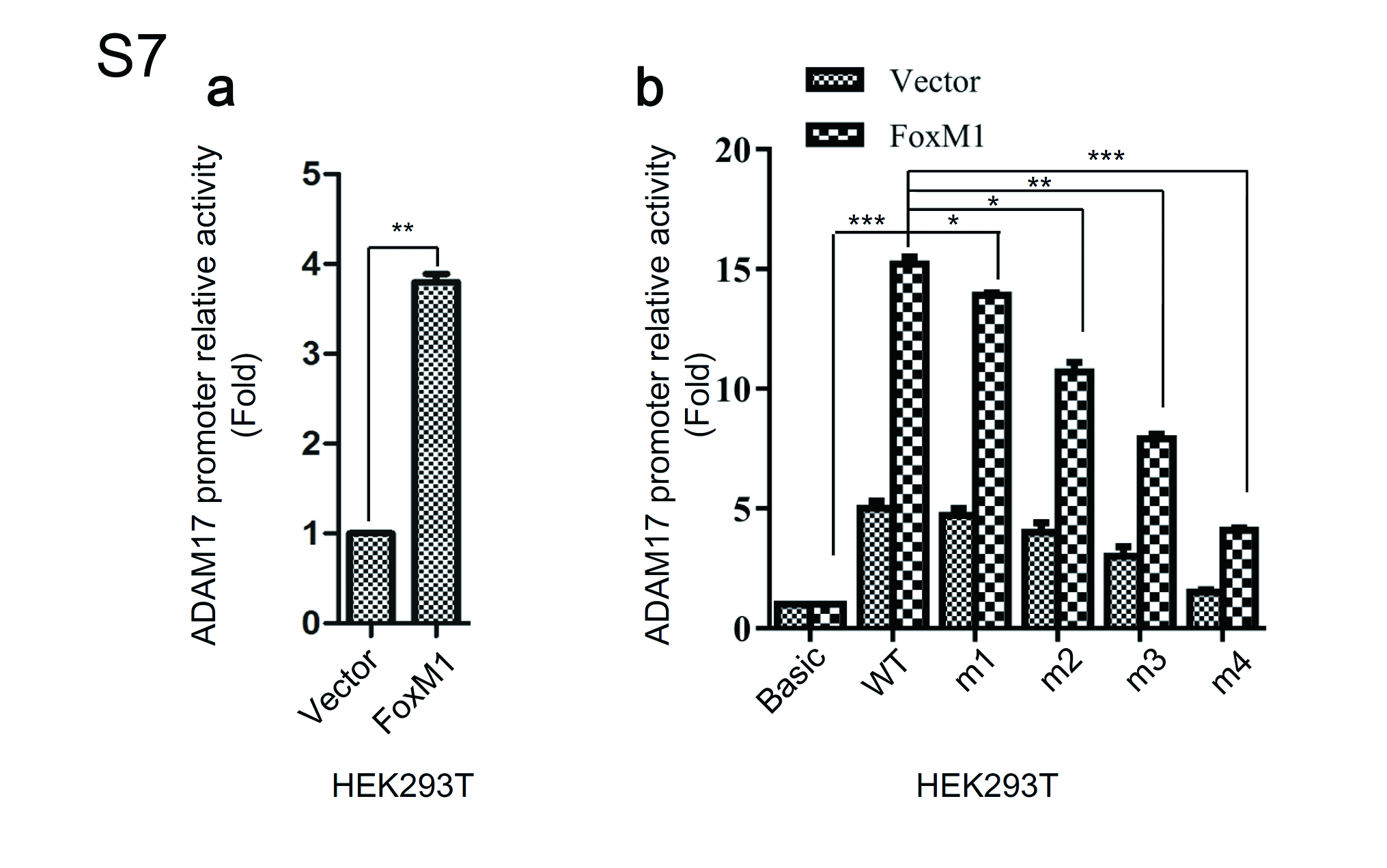

Supplement: Supplementary file 7 — Figure S7 [file 41419_2018_482_MOESM7_ESM.tif]

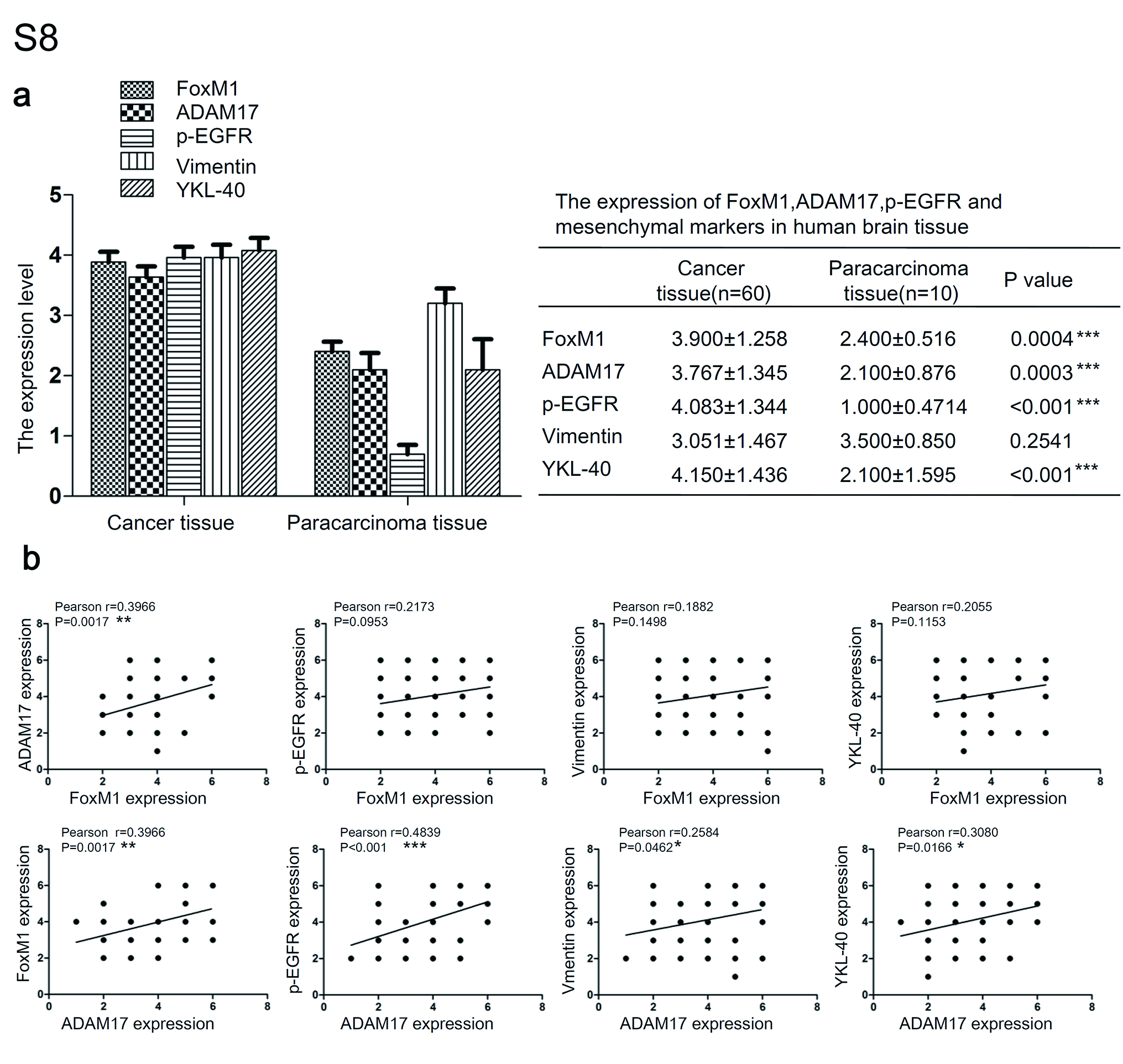

Supplement: Supplementary file 8 — Figure S8 [file 41419_2018_482_MOESM8_ESM.tif]
