# Supplementary material for: FoxM1 drives ADAM17/EGFR activation loop to promote mesenchymal transition in glioblastoma
Source: Cell Death Dis. 2018 Apr 27;9(5):469. doi: 10.1038/s41419-018-0482-4 (PMC5920065; doi:10.1038/s41419-018-0482-4)
Supplement: Supplementary file 9 — Supplementary figure legends -CDDis-revised [file 41419_2018_482_MOESM9_ESM.docx]

**Figure.S1. The expression levels of FoxM1 and ADAM17 are positively correlated with mesenchymal markers in glioma cells**. **a** The correlation of FoxM1 and ADAM17 with mesenchymal marker gene expression was analyzed and presented by correlation analysis showing the correlation coefficient by making use of the platform: https://hgserver1.amc.nl/cgibin/r2/main.cgi. **b** Kaplan-Meier algorithm was performed to evaluate overall survival time between FoxM1 high and low expression groups, as well as ADAM17. **c** FoxM1, ADAM17 and mesenchymal markers expression in glioma cells were detected by qRT-PCR. Data are shown as the mean ±s.d. of three independent experiments. Student’s t-test was used to determine the significance of the differences between the groups (^#^P<0.0001, Student’s t-test)**. d**

The expression of FoxM1, ADAM17 and mesenchymal markers in SW1783 and U87MG cells were detected by immunofluorescence analyses. Scale bar=100μm.

**Figure.S2**. **The transfection efficiency was confirmed after transfection. a-b** qRT-PCR and western blot were used to confirm FoxM1 knockdown or overexpression in glioma cells. β-Tubulin was used as a loading control. **c-d** qRT-PCR and western blot were used to confirm ADAM17 knockdown or overexpression in glioma cells. Data are shown as the mean ±s.d. of three independent experiments. Student’s t-test was used to determine the significance of the differences between the groups (^#^P<0.0001, Student’s t-test)**.**

**Figure.S3-4. Quantification of western blot**

Quantification of the western blots was performed using [Image J Software](http://xueshu.baidu.com/s?wd=paperuri%3A%288d438da4ccc5690128c0915b2d3ce2d3%29&filter=sc_long_sign&sc_ks_para=q%3DA%20method%20to%20exactly%20measure%20the%20morphological%20quantity%20of%20leaf%20using%20Scanner%20and%20Image%20J%20Software.&sc_us=9181668815539152210&tn=SE_baiduxueshu_c1gjeupa&ie=utf-8). Data are shown as the mean ±s.d. of three independent experiments. Student’s t-test was used to determine the significance of the differences between the groups (*P<0.05, Student’s t-test).

**Figure.S5. Exogenous FoxM1 or ADAM17 induced mesenchymal transition in glioma cells. a-b** FoxM1 overexpression increased the migration capacities as well as potentialities of adipogenesis and osteogenesis of SW1783 and LN229 cells. Scale bar=100μm. Data are shown as the mean ±s.d. of three independent experiments. Student’s t-test was used to determine the significance of the differences between the groups (***P<0.001, ^#^P<0.0001, Student’s t-test)**.** **c** The expression of mesenchymal markers and E-cadherin were detected after overexpressing FoxM1 by western blot. **d-e** ADAM17 overexpression enhanced the abilities of migration, adipogenesis and osteogenesis in SW1783 and LN229 cells. Scale bar=100μm. Data are shown as the mean ±s.d. of three independent experiments. Student’s t-test was used to determine the significance of the differences between the groups (***P<0.001, Student’s t-test)**.** **f** The expression of mesenchymal markers and E-cadherin were detected after overexpressing ADAM17 by western blot.

**Figure.S6. The mRNA levels of FoxM1were detected after upregulating ADAM17.** ADAM17 had no effects on the mRNA levels of FoxM1 in SW1783 and LN229 cells. Data are shown as the mean ±s.d. of three independent experiments. Student’s t-test was used to determine the significance of the differences between the groups ( ^#^P<0.0001, Student’s t-test)**.**

**Figure.S7. FoxM1 transcriptionally promotes ADAM17 expression through binding to the promoter of ADAM17**.

**a** FoxM1 overexpression increased the ADAM17 promotor activity in HEK293T cells. Data are shown as the mean ±s.d. of three independent experiments. Student’s t-test was used to determine the significance of the differences between the groups (*P<0.05, Student’s t-test). **b** The luciferase report assay was used to determine the importance of the binding sites on ADAM17 promoter activity in HEK293T cells. Data are shown as the mean ±s.d. of three independent experiments. Student’s t-test was used to determine the significance of the differences between the groups (*P<0.05, Student’s t-test).

**Figure.S8. The correlation among the expression of FoxM1, ADAM17 and mesenchymal markers were detected by IHC. a** Quantification of staining was performed to compare the protein levels between cancer tissues and normal brain. *P<0.05. **b** Linear regression analyses were conducted to detect the correlation of FoxM1, ADAM17 and mesenchymal markers expression. Data are shown as the mean ±s.d. of three independent experiments. Student’s t-test was used to determine the significance of the differences between the groups (*P<0.05, ***P<0.05, Student’s t-test)**.**
