# Supplementary material for: FoxM1 drives ADAM17/EGFR activation loop to promote mesenchymal transition in glioblastoma
Source: Cell Death Dis. 2018 Apr 27;9(5):469. doi: 10.1038/s41419-018-0482-4 (PMC5920065; doi:10.1038/s41419-018-0482-4)
Supplement: Supplementary file 10 — Supplementary tables [file 41419_2018_482_MOESM10_ESM.docx]

| Table 1. The primers for PCR | |
| --- | --- |
| Name | Sequence(5’ to 3’) |
| FoxM1 RT-Forward | TGGACCAGGTGTTTAAGCAGC |
| FoxM1 RT-Reverse | GGGAGTTCGGTTTTGATGGTC |
| ADAM17 RT-Forward | TTATTGGTGGTAGCAGAT |
| ADAM17 RT-Reverse | AAGTGTTCCGATAGATGT |
| vimentin RT-Forward | CGGGAGAAATTGCAGGAGGA |
| vimentin RT-Reverse | AAGGTCAAGACGTGCCAGAG |
| YKL-40 RT-Forward | TCCAGTGCTGCTCTGCATAC |
| YKL-40 RT-Reverse | CCAGGTGTCGATGTGATCGT |
| GAPDH RT-Forward | GCTCAGAACACCTATGGGGA |
| GAPDH RT-Reverse | AAGTGTTCCGATAGATGT |
| ADAM17 promoter CHIP-Forward | AATGGACCAAGTATAGGATT |
| ADAM17 promoter CHIP- Reverse | GCAGACACTTCAACAAAT |

| Table 2. shRNA target sequences and mutant primer sequences | |
| --- | --- |
| Name | Sequence(5’ to 3’) |
| target sequences of sh-EGFP | TACAACAGCCACAA CGTCTAT |
| target sequences of sh-FoxM1 | GCCAATCGTTCTCTGACAGAA |
| target sequences of sh-ADAM17 | CCTATGTCGATGCTGAACAAA |
| ADAM17 promoter Forward | AATGGACCAAGTATAGGATT |
| ADAM17 promoter Reverse | GCAGACACTTCAACAAAT |
| ADAM17 m1 Forward | GCATAAAACAGCAACATGTACCCAACGT |
| ADAM17 m1 Reverse | AGTTCAACCAAAGTTTATAAAAGTAGGACATAAT |
| ADAM17 m2 Forward | GCCATTCACTAAACTATACAGAGCT |
| ADAM17 m2 Reverse | TATGGTACTGAATAAAACAGTCTAAAAAC |
| ADAM17 m3 Forward | TATGGTACTGAATAAAACAGTCTAAAAAC |
| ADAM17 m3 Reverse | TATGGTACTGAATAAAACAGTCTAAAAAC |
| ADAM17 m4 Forward | GGGGCGTGGAGCAAATGTGCATT |
| ADAM17 m4 Reverse | TGCTGTAGGGAGAGGGTCTGCAGACACT |
